# Emerging Technologies for Percutaneous Mitral Valve Repair

**DOI:** 10.3389/fcvm.2019.00161

**Published:** 2019-11-06

**Authors:** Antonio Mangieri, Alessandra Laricchia, Francesco Giannini, Francesco Gallo, Faraj Kargoli, Annamaria Ladanyi, Luca Testa, Antonio Colombo, Azeem Latib

**Affiliations:** ^1^GVM Care and Research, Maria Cecilia Hospital, Ravenna, Italy; ^2^Division of Cardiology, Department of Medicine, Montefiore Medical Center, Bronx, NY, United States; ^3^San Raffaele University Hospital, Milan, Italy; ^4^Department of Clinical and Interventional Cardiology, IRCCS Policlinico San Donato, Milan, Italy; ^5^Division of Cardiology, Department of Medicine, University of Cape Town, Cape Town, South Africa

**Keywords:** mitral regurgitation, heart failure, mitral valve, transcatheter mitral valve replacement, mitral annulus

## Abstract

Mitral regurgitation (MR) is a common disease affecting more than 4 million people in the United States and the European Union. A significant number of percutaneous valves have been developed recently, specifically designed for the mitral anatomy, and with a promising evidence of good procedural and echocardiographic outcomes. However, even if transcatheter mitral valve replacement (TMVR) will have a role in the future of percutaneous treatment of both functional and degenerative mitral regurgitation, percutaneous mitral valve repair will always play a vital role in the treatment of MR because of the favorable safety profile and the fact that it respects the native anatomy. In this review, we will discuss the new emerging technologies under development to treat mitral regurgitation focusing on different devices that aim to target different components of the mitral anatomy.

## Introduction

Mitral regurgitation (MR) affects more than 4 million people in the United States and the European Union and its prevalence increases with age, reaching up to 1 in 10 adults aged 75 years or older (1, 2). Degenerative MR (DMR) accounts for approximately one-third of all MR cases (3). Today, surgical mitral valve repair (MVr) is a robust and effective procedure to correct MR, with years of clinical experience and validated evidence. However, surgical mitral intervention in high risk patients is still a challenging procedure, with 30-day mortality approaching 3.1% (4). Furthermore, the mortality rate is even higher in patients with functional mitral regurgitation (FMR), where a concomitant impairment of the left ventricular ejection fraction (LVEF) is often observed (5).

For these reasons, emerging low risk percutaneous strategies are needed to treat MR in both degenerative and functional anatomies, and to minimize the potential complications associated with open-heart surgery. Percutaneous MVr technologies are required to replicate surgical mitral valve reconstruction, without disrupting the normal valve and ventricular physiology. In this review, we will discuss the potential future role of percutaneous MVr, offering an overview on the emerging technologies that are currently under investigation.

## Is the Future Still Repair?

A variety of repair techniques (including mitral leaflet devices, implantation of neochords and percutaneous mitral annuloplasty) has been introduced since the first MitraClip procedure in 2003. However, in most of these cases, emerging technologies have been dismissed because of suboptimal pre-clinical results, complicated implants, and difficult reproducibility of their results. The development of transcatheter MVr systems is facing the following challenges:

1. The degree of MR reduction with percutaneous repair technologies is not fully predictable, and a complete resolution is rarely guaranteed.
2. Mitral valve anatomy is complex and different mechanisms can contribute to regurgitation. As a consequence, a single repair device addressing a single target will unlikely achieve optimal results. For example, a certain degree of mitral annular dilatation is always present in patients suffering from severe MR. Surgical MVr usually combines various types of leaflet-plasty with annular reduction in order to minimize the risk of progressive annular enlargement and MR recurrence (6). On the contrary, percutaneous MVr procedures are often based on a single repair technique and are consequently considered “incomplete.” The lack of knowledge on the long-term outcomes in these circumstances gives rise to concerns about the durability of MR reduction. Isolated published case reports show the feasibility of simultaneous implantation of MitraClip (Abbott Laboratories, Abbott Park, IL) and Cardioband (Edwards Lifesciences Corp., Irvine, CA, USA) as well as “rescue” percutaneous annuloplasty to treat MR recurrence after Mitraclip (7, 8). However, these still remain anecdotal cases and more data are needed.
3. The mitral annulus is a highly dynamic, asymmetrical structure, with a saddle-shape conformation that poses difficulties in the positioning and sizing of these devices. Therefore, the development of percutaneous annuloplasty systems has to overcome major design obstacles due to the aforementioned complex mitral annular structure. A circular prosthetic ring runs the risk of not matching the asymmetrical anatomy of the native mitral valve annulus, thus increasing the tension between the device and the mitral structure. This limitation along with the method of fixation (screws) could translate into an increased risk of late device detachment, which has already been observed in the early cases of percutaneous mitral annuloplasty (9).
4. A certain proportion of mitral anatomies are not the ideal candidates for repair and sometimes TMVR can be an alternative treatment option. Nevertheless, calcific and rheumatic MR still represent important unmet needs in transcatheter mitral intervention that may be associated with suboptimal results, both for repair (increased risk of post-procedural gradients) and replacement (due to the increased risk of para-valvular leakage and left ventricular outflow tract obstruction).

### The Advantages of Repair Over Replacement

Taking into consideration the aforementioned challenges, a significant number of percutaneous valves have been recently developed, specifically designed for the mitral anatomy, and with promising evidence of good procedural and echocardiographic outcomes. However, even if TMVR will have a definite role in the future of percutaneous treatment of both FMR and DMR, percutaneous MVr will always play a vital role in the treatment of MR due to the following:

1. ***Technical aspects:*** Transcatheter valves and percutaneous annuloplasty devices have to adapt to the mitral annulus that, conversely to the fibrous ring of the aortic valve, does not provide a rigid anchor for sealing. Moreover, the mitral annulus is posteriorly embedded in the junction of the left atrium and left ventricle, while the anterior portion consists of the aorto-mitral curtain, a dynamic structure with limited rigidity. This poses multiple challenges for securing a device and bares the risk of compression and interference with the aortic valve apparatus. Data from the preliminary experience of these devices demonstrates that the risk of complications like valve erosion, migration, malposition at 1-year follow-up is around 4% (10). These complications theoretically do not exist in the case of MVr.
  The close proximity of the left ventricular outflow tract (LVOT) places it at a higher risk for obstruction, especially with high-profile valves. In one study on the early experience with TMVR, the rate of acute LVOT obstruction was 8.2% with transcatheter mitral valve-in-ring (ViR) procedures, and 9.3% following TMVR in the presence of severe mitral annular calcification (MAC) (11, 12). Acute LVOT obstruction has a negative impact on both, procedural and post-procedural outcomes. A neo-LVOT area of <1.7 cm^2^ is highly predictive of obstruction, and patients with this anatomy should be considered for MVr rather than replacement (13). LVOT obstruction is not a problem with percutaneous MVr, since the devices implemented for percutaneous mitral valve plasty are much smaller in size, and have a favorable profile that respects the mitral valve anatomy.
  Lastly, the anatomical position of the mitral valve apparatus can be easily reached through a transapical approach. However, in sick patients with low ejection fraction, the presence of apical scar, and thinning of the apical wall are deleterious, thus an alternative access would be preferable to minimize the risk of complications. A fully percutaneous transfemoral trans-septal venous approach would be desirable but, in comparison to MVr, it would imply larger iatrogenic interatrial defects and it would require a highly flexible delivery system to coaxially reach the mitral valve plane (14).
2. ***Durability:*** Although no long-term durability data exist for TMVR, we know from surgery that structural valve degeneration occurs more frequently in mitral bioprosthetic valves than in the aortic valves, and in younger individuals (15). From our clinical experience we also learned that patients undergoing TMVR are younger and have a longer life expectancy compared to transcatheter aortic valve implantation (TAVI) patients. All these reasons raise the alarms regarding the long-term durability of these valves. Conversely, the devices used for MVr can potentially last for decades without a concrete risk of erosion or degeneration.
3. ***Device thrombosis:*** TMVR prostheses are potentially more prone to thrombosis due to the larger size, the high profile and the huge amount of “foreign” material of which they are made. Moreover, the atrial aspect of the mitral prostheses is exposed to low atrial pressures that can contribute to blood stasis, and subsequently to valve thrombosis. The early experience in 100 patients reported a device thrombosis rate of 6% at 1-year (16). Conversely, percutaneous MVr is more physiological as the implanted device is not as bulky as TMVR and the smaller surface area can mitigate the risk of device thrombosis.
4. ***Paravalvular Leak (PVL):*** Paravalvular leak (PVL) is a common complication of mitral valve replacement (15). Although most PVLs have unknown clinical significance, ~3% of patients will have signs and symptoms of hemolysis, heart failure or a combination of the two (17). Significant PVL is relatively rare in cases of TMVR in non-calcified mitral annuli, while in cases of TMVR in MAC, the rate of moderate to severe PVL at 30 days can reach up to 13.8% (18). In MVr the risk of PVL leading to hemolysis is theoretically non-existent.

## Transcatheter Mitral Valve Repair: What Can We Expect From the Future

MR is the most frequent valve disease in the population and it's prevalence increases with age (19). Open-heart surgery is considered the gold standard for the treatment of severe MR, with excellent outcomes achievable in most patients. However, more than 50% of patients with severe MR are excluded from surgery due to an increased perioperative risk related to comorbidities (20). In particular, patients with FMR, have a high perioperative mortality ranging between 6.6 and 11.4% (21). Whereas, the use of an effective percutaneous MVr system can result in a lower perioperative risk with similar clinical benefits compared to surgery at follow-up. Lastly, we hypothesize that the number of future percutaneous mitral valve procedures will be influenced by the following factors:

- The results of the recently published COAPT and MITRA-FR trials have demonstrated that moderate to severe FMR has a clear clinical impact on prognosis, and a successful treatment of FMR with the MitraClip system in selected patients can significantly reduce the rate of rehospitalization, and all-cause mortality at 2-years follow-up (22, 23).
- The prevalence of heart failure will increase by ~50% between 2012 and 2030, resulting in more than 8 million people older than 18 years-old with heart failure. This daunting future reflects the increased prevalence of heart failure as the population ages, and the improved survival of patients with acute myocardial infarction and heart failure itself. In parallel, this progressive increase of heart failure prevalence will translate into a higher rate of MR. Hence, percutaneous treatments to fix MR will be necessary given the high surgical risk profile of this population (24).
- In the near future, the threshold for percutaneous treatment of multiple valvular diseases will be lowered. TAVI has already been demonstrated to be non-inferior or even superior to surgical aortic valve replacement in low risk patients. Percutaneous treatment of concomitant significant MR in this population will be considered a desirable option (25, 26).
- The armamentarium of devices for the treatment of MR will expand, offering a wide number of percutaneous options that will be able to accommodate a larger variety of anatomies.

## Emerging Devices

**Figure 1:**
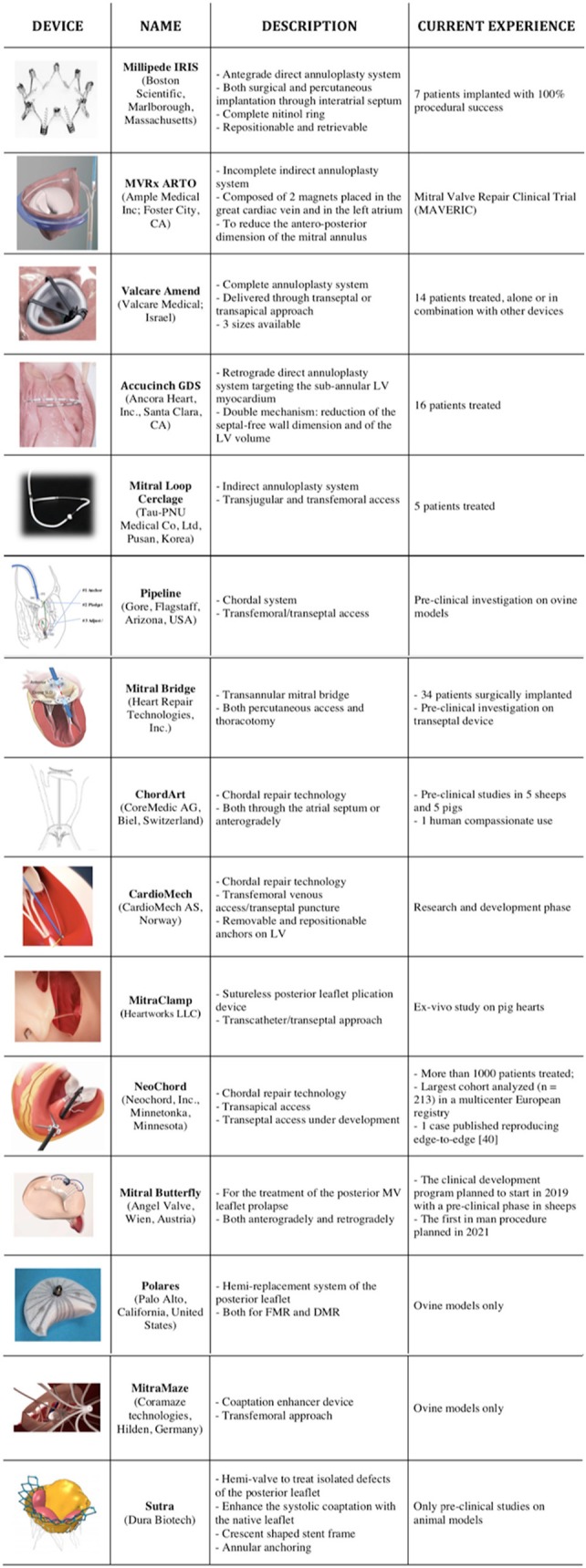
List of the emerging repair technologies available for mitral valve.

A wide number of MVr systems are currently under development (Figure 1). The following section provides a brief overview of these devices and of the ongoing clinical studies on transcatheter mitral valve repair (Table 1).

**Table 1:** Ongoing trials for each mitral valve repair system.

**Device name**	**Ongoing trials**	**Description**
Millipede IRIS	**NCT02607527:** The annular reshaping of the mitral valve for MR using the millipede IRIS system Recruiting	Enrollment period: January 2017/2020 Multicenter Single group assignment Estimated 50 patients Primary endpoint: acute safety Secondary endpoint: efficacy
MVRx ARTO	**NCT02302872** (MAVERIC EU): Treatment of heart failure and associated functional mitral valve regurgitation Recruiting	Enrollment period: October 2013/May 2020 Single group assignment Estimated 45 patients Primary endpoints: MAE at 30-days, MR grade and change from baseline at 30-days, device technical success
	**NCT03311295** (MAVERIC US): MitrAl valve repair clinical trial—United States Recruiting	Enrollment period: April 2018/2024 Single group assignment Estimated 15 patients Primary endpoints: safety at 30-days, efficacy
Valcare amend	**NCT02602613** (AMENDTM trial): mitral valve repair system, annuloplasty ring applied in a transcatheter method Recruiting	Enrollment period: December 2015/2018 Multicenter Single group assignment 40 patients Primary endpoints: safety at 30-days, device technical success
Accucinch GDS	**NCT03183895** (CorCinch-EU Study): safety and performance evaluation of the accucinch ventricular repair system for functional mitral regurgitation due to dilated ischemic or non-ischemic cardiomyopathy Not yet recruiting	Enrollment period: February 2019/January 2023 Multicenter Single group assignment Estimated 132 patients Primary endpoint: safety at 30-days Secondary endpoints: technical success, structural performance, freedom from re-hospitalizations or re-interventions, improvement in status
	**NCT03560167** (CorCinch-PMVI Study): an early feasibility study of the accucinch ventricular repair system in patients with prior mitral valve intervention (PMVI) and recurrent mitral regurgitation Recruiting	Enrollment period: June 2018/September 2024 Multicenter Single group assignment Estimated 15 patients Primary endpoint: device-related or procedure-related MAE
Mitral loop cerclage	**NCT03453853** (AFRICA Study): atrial functional mitral regurgitation response in mitral loop cerclage annuloplasty Recruiting	Enrollment period: April 2018/March 2020 Multicenter Single group assignment Estimated 5 patients Primary endpoint: change of mitral regurgitation severity/mitral annulus geometry at 30-days, safety at 30-days
Mitral bridge	**NCT03511716**: HRT observational study of a mitral bridge in patients with moderate to severe mitral valve regurgitation to evaluate device safety and performance Active, not recruiting	Enrollment period: February 2014/July 2020 Multicenter Observational 34 patients Primary endpoint: freedom from subsequent open mitral valve repair or replacement
ChordArt	**NCT03581656** (CHAGALL): ChordArt System Study for the Treatment of Mitral Regurgitation Due to Leaflet Prolapse or Flail Recruiting	Enrollment period: March 2018/July 2020 Multicenter Single group assignment Estimated 40 patients Primary endpoint: all cause mortality/MAE at 30-days Secondary endpoint: technical success, device performance at 30-days
NeoChord	**NCT02803957** (ReChord): Randomized Trial of the NeoChord™ DS1000™ System Versus Open Surgical Repair Recruiting	Enrollment period: November 2016/July2025 Multicenter Randomized trial Estimated 585 patients Primary endpoints: freedom from MAE/ grade II, III or IV MR, mitral valve replacement or mitral valve reintervention at 30-days

*MAE, major adverse event; MR, mitral regurgitation; NYHA, New York Heart Association; 6MWT, 6-min walk test; KCCQ-QoL, Kansas City Cardiomyopathy Questionnaire for Quality of Life; LV, left ventricle*.

### Millipede IRIS

The Millipede IRIS is a complete, semi-rigid annuloplasty ring (Boston Scientific, Marlborough, Massachusetts). The system is composed of eight stainless-steel anchors that are connected by sliding collars and nitinol frames, located in the upper position of the device. Each anchor can be independently and reversibly fixed to the mitral tissue in order to customize the size and the site of anchoring. The Millipede IRIS has been implanted both surgically, using a trans-atrial approach, and percutaneously, with a 24 French steerable catheter delivered trans-septally. The first clinical experience with IRIS included seven patients: four were treated using a conventional surgical approach, while three patients were treated percutaneously. The percutaneous implant of the device was monitored under fluoroscopy and using both transesophageal and intracardial ultrasound (ICE) monitoring. The adjunctive use of ICE allowed a direct visualization of the mitral annulus and a detailed placement of the IRIS anchors. All the iatrogenic atrial septal defects were closed with a 10 mm Amplatzer septal occluders (Abbott, Santa Clara, CA). The authors reported no major adverse events and a procedural success obtained in all the cases. The implant resulted in a significant reduction in the septo-lateral diameter (from 38.0 ± 4.1 to 25.9 ± 4.9 mm), and in a significant reverse remodeling of the left ventricle, with a decrease in diastolic left ventricular volumes from 182.4 ± 54.3 to 115.3 ± 98.8 mL at 30 days. Every patient demonstrated reduction of MR, with all patients showing a decline from a baseline of 3 or 4+ MR to 0 or 1+ MR at 30 days (27). One of the patients who received a surgical implantation of the IRIS Millipede in combination with an A2/P2 Alfieri leaflet repair, performed in standard fashion, experienced a relapse of MR at 12 months follow-up due to a newly ruptured chordae tendineae, just lateral to the original Alfieri stitch. In this patient, an adjunctive MitraClip was placed with a residual trivial MR (28).

The Annular Reshaping of the Mitral Valve for MR Using the Millipede IRIS System (NCT02607527) is an early feasibility clinical trial with the aim of enrolling 50 patients with symptomatic severe MR treated percutaneously with the IRIS device.

### MVRx ARTO

The MVRx ARTO (Ample Medical Inc.; Foster City, CA) is an incomplete and indirect annuloplasty system. The system is composed of two magnets (MagneCath) that are placed in the great cardiac vein and in the left atrium at the level of A2/P2 scallops. Once the two magnets are close to each-other, a specific guidewire is passed through the two MagneCath from the great cardiac vein to the left atrium, aligning the two catheters. After using an exchange catheter, the loop guidewire is placed across left atrium. This guidewire directs the placement of the great cardiac vein anchor (T-bar) and septal anchor. Once in place, an appropriate tension is applied to reduce the antero-posterior dimension of the mitral annulus. The results of the Mitral Valve Repair Clinical Trial (MAVERIC) have been published, where the authors reported the 30-days outcome of 11 patients that were treated with the MVRx ARTO system. Effective regurgitant orifice area decreased from 30.3 ± 11.1 to 13.5 ± 7.1 mm^2^, and regurgitant volumes from 45.4 ± 15.0 to 19.5 ± 10.2 ml. The mitral annular anteroposterior diameter decreased from 45.0 ± 3.3 to 38.7 ± 3.0 mm. One patient had pericardial effusion, and one asymptomatic device dislodgment were reported at 30-days, with no other major adverse events (29). To evaluate the safety and efficacy of this system in patients with MR secondary to annular dilatation, the MAVERIC EU (NCT02302872) and the MAVERIC US (NCT03311295) clinical trials are recruiting 45 and 15 patients in Europe and the United States, respectively. A total of 45 patients have been recruited in both trials with 1-year echocardiographic follow-up available in 35 patients. The authors report a significant reduction of the mitral annular dimensions from 41.4 to 35.3 mm, sustained at 1-year, with a freedom from residual moderate MR obtained in 92% of patients. At the follow-up, 11.4% of patients died for cardiovascular reasons, with no device malfunctioning reported (30).

### Valcare Amend

Valcare Amend system (Valcare Medical; Israel) is a complete, semi-rigid D-shaped percutaneous annuloplasty system. The ring is available in three sizes to fit a wide range of mitral annular dimensions (29–50 mm). Once in place, the ring is delivered starting from the posterior part of the annulus. The annuloplasty ring is designed to be delivered through a transseptal or a trans-apical approach. This annuloplasty system has been tested in different scenarios and in combination with other therapies. A total of 14 patients have been treated in the first clinical experience: eight patients with FMR have received Valcare Amend as a single therapy. Whereas, two patients with DMR have received Valcare Amend as a single therapy. In another four patients, the system has been utilized in combination with MitraClip (three patients) and with NeoChord (one patient). The implant resulted in a 74% reduction of the jet area and in a 20% reduction in the antero-posterior diameter (31). In a single case, an unplanned MitraClip was implanted to treat significant residual mitral regurgitation. The Mitral Valve Repair System, Annuloplasty Ring Applied in a Transcatheter Method (AMENDTM trial, NCT02602613) is currently recruiting with a target sample size of 40 patients to evaluate the efficacy and safety of the device.

### Accucinch GDS

The Accucinch GDS device (Ancora Heart, Inc., Santa Clara, CA) is a direct ventriculoplasty system targeting the sub-annular left ventricular myocardium. The system includes a delivery catheter, a modular guide tunnel (MGT), an anchor delivery catheter and 12–16 nitinol anchors, connected by an ultra-high molecular weight polyethylene cinch cable to exercise tension over them. Proximal and distal anchors are interspaced with nitinol force distribution members. The system is implanted retrogradely using an 18 French guiding catheter delivered through the aortic valve. The Accucinch GDS implant can be customized according to the sub-annular space anatomy and to the thickness of the left ventricular wall. Anchors are delivered from commissure to commissure through an arch beneath the posterior leaflet of the MV in the ventricular free wall. Once the system has reached the sub-annular space, the MGT is oriented in direction of the myocardium and gradually withdrawn to facilitate anchor delivery through an inner tunnel with a single window. Once all the anchors have been released, tension is applied to the cinch cable using the cinch and lock catheter. A cut catheter is then utilized to cut the cinch cable before removing the MGT and the guide catheter (32). Thanks to the implantation site, the Accucinch GDS device acts with a double mechanism; it reduces the septal-free wall dimension, drawing the papillary muscles and the mitral leaflets in close proximity, and reduces the left ventricle volume without extracting muscle. Compared to the first implants, the latest version of the device is more flexible. Moreover, to achieve a greater volumetric reduction, the target zone now includes a wider part of left ventricular free wall. The Accucinch GDS has been attempted in 16 patients with two failures due to anatomical constraints and impossibility to achieve an adequate position of the delivery catheter. Among 14 successful implants, one procedure was complicated by pericardial effusion and stroke, that lead to the patient's demise. The 6 month echocardiographic data showed a significant reduction of the mitral regurgitant volume (from 60 to 37 ml) together with a sustained reduction of the left ventricular end-systolic volume (from 119 to 72 ml), with a continuous trend toward a progressive reverse remodeling of the left ventricle. The efficacy and safety of the device is under investigation in different scenarios (33). TheCorCinch-EU Study (NCT03183895) is an international multicenter, non-randomized, prospective safety study, designed to evaluate the AccuCinch Ventricular Repair System for the treatment of heart failure, with or without FMR due to dilated ischemic or non-ischemic cardiomyopathy. The Early Feasibility Study of the AccuCinch Ventricular Repair System in Patients With Prior Mitral Valve Intervention (PMVI) and Recurrent Mitral Regurgitation (The CorCinch-PMVI Study, NCT03560167) is actively recruiting, with target sample size of 15 patients with recurrent MR after failure of the previous valve intervention.

### Mitral Loop Cerclage

The Transmural System Transcatheter Mitral Cerclage Annuloplasty (Tau-PNU Medical Co. Ltd., Pusan, Korea) is an indirect annuloplasty system composed of a stainless steel tension element with a coronary artery protection system. The two extremities of the tension element are connected using a bridge device that extends to the left subclavian vein. An adjustable extravascular lock is fixed subcutaneously in the subclavicular fossa and has the function to connect the tension element and the bridge, thus allowing a modulation of the tension under echocardiographic monitoring (34).

The procedure is performed using both the transjugular and transfemoral approach. Once the coronary sinus has been engaged, a pressurized venogram is performed to identify a perforator vein suitable for the intervention. A guidewire is then advanced in the perforator vein and externalized into the right ventricular outflow tract, passing through the interventricular septum. The externalized guidewire is then snared and pulled back into the inferior vein cava. The guidewire is subsequently exchanged with the Mitral Loop Tension device that is deployed with a protection device, to prevent the extrinsic compression of the circumflex artery. Once the tension device is *in situ*, the bifid coronary sinus tricuspid valve bridge is placed to prevent any damage of the septal tricuspid leaflet and of the conduction system. The first in human experience with the mitral loop cerclage annuloplasty was successful in 4 of 5 attempts. The mitral regurgitant volume and the septo-lateral mitral annular diameter were significantly reduced at 6 months (54 ± 8–18.5 ± 4.1 ml and 41.5–34.2 mm, respectively). The implantation of the mitral Cerclage also lead to a reduction of the left ventricular end diastolic volume (140 ± 62.5–102.6 ± 35.7 ml) (35). The Atrial Functional Mitral Regurgitation Response In Mitral Loop Cerclage Annuloplasty (AFRICA Study, NCT03453853) is a prospective, single-center, open label, feasibility study to assess the safety and efficacy of the Mitral Loop Cerclage Annuloplasty in treating FMR associated with heart failure and atrial fibrillation.

### Pipeline

The Pipeline system (Gore, Flagstaff, Arizona, USA) is a newly developed system that targets the sub-annular apparatus, and acts as a chordal system that can be implanted using a transfemoral trans-septal approach. Once the delivery catheter reaches the left ventricle, a ventricular anchor is deployed from the catheter and fixed to the free wall, leaving a ventricular suture attached to the ventricular anchor. A leaflet pledget is deployed to secure the mitral valve leaflet to the ventricular anchor. The leaflet suture is secured to the ventricular anchor to limit the excursion the leaflet. This technology is currently under pre-clinical investigation with mid-term ovine models showing the feasibility of the procedure.

### Mitral Bridge

Mitral Bridge (Heart Repair Technologies, Morgan Hill, CA, USA) is a newly developed technology that consists in a curvilinear nitinol band covered by a silicone overmold with velour pads at either extremity. The Mitral Bridge is positioned transversely across the mitral valve, linking the anterior and the posterior leaflets at the level of A2-P2 segments, thus reducing the antero-posterior annular dimensions. A delivery handle, preattached to the implant, assists in positioning the bridge on the annulus with the curvature facing the ventricular cavity. Five septal-laterally oriented sizes of the mitral bridge (22, 24, 26, 28, and 30 mm) are available. The initial experience included 34 patients enrolled in the observational study of the Heart Repair Technologies Mitral Bridge in Treating Mitral Valve Regurgitation (NCT03511716), who received a surgical implant of the Mitral Bridge. At 2 years, no strokes or device-related adverse events were noted, and the MR was reduced from 3.32 ± 0.47 to 0.50 ± 0.83 (*P* < 0.001), with <1+ MR in 33/34 patients (including four reinterventions for periprosthetic recurrent MR ≥3 without mitral bridge explants or conventional mitral repair or replacement). At 2 years, the mean mitral gradient was 2.15 ± 0.82 mmHg, and the mitral annular septo-lateral dimension decreased from 40.4 ± 2.91 to 28.9 ± 1.55 mm (36).

### ChordArt

The ChordArt *(CoreMedic AG, Biel, Switzerland)* is a fully percutaneous transcatheter mitral chordae implantation system, which can be delivered through the transtrially or transfemorally via the trans-septal approach. ChordArt uses a dedicated delivery catheter that grasps the mitral leaflet in the target area that needs to be treated. Once grasped, the delivery system is passed through the punctured leaflet until it reaches the papillary muscle. Once at the level of the papillary muscle, an anchor located at the distal tip of the catheter is fixed into the muscle. After this maneuver, the delivery system is retrieved leaving a suture that connects the grasped leaflet and the subvalvular apparatus. Pre-clinical studies have evaluated the safety and the efficacy of the system, surgically implanted in the beating heart of five sheep with acute chordal rupture; all procedures were successful and all of the five animal models were alive at 6 months follow-up, with no evidence of disruption or malfunction of the device. The ChordArt system has been subsequently implanted also in five pigs with acute mitral valve chordal rupture. The device has been implanted under fluoroscopy and echocardiographic guidance using left thoracotomy, with direct access through the left atrium on a beating heart. After the positive first in human experience for compassionate use, the device performance and the technical efficacy of ChordArt is currently under investigation in the ChordArt System for Mitral Regurgitation trial (CHAGALL, NCT03581656) with the planned enrollment of 40 patients suffering from a flail or prolapsed mitral valve leaflet (37).

### CardioMech

CardioMech (CardioMech AS, Norway) is a transcatheter mitral valve chordal repair technology designed for the treatment of DMR due to prolapsed or flail leaflets. It is still in the research and developmental phase. It requires a transfemoral venous access and a transseptal puncture to reach the left atrium. The device is advanced through a 24Fr steerable delivery catheter to grip the prolapsing leaflet, then the new chordae is attached from the leaflet to the ventricular wall, where an anchor fixes it. Anchors are removable and repositionable. Studies to assess safety and feasibility are needed.

### MitraClamp

MitraClamp *(Heartworks LLC)* is a new sutureless leaflet plication device designed for treating patients with mitral leaflet prolapse through a transcatheter approach. The U-shape arms are able to rotate around a common axis. Following leaflet grasping, leaflet plication is performed by rotating the two arms in two opposite ways. Pre-clinical study on the application of the MitraClamp in six fresh pig hearts demonstrated a dramatic reduction of the regurgitant volume during hydrodynamic tests. Device anchorage to leaflets was also found to be stable after device locking (38).

### Neochord

The NeoChord Artificial Chordae Delivery System (Neochord, Inc., Minnetonka, Minnesota) is a transcatheter MVr technology performed through a transapical access (a transseptal access system is still under development). Under general anesthesia and transesophageal echo guidance, it allows the placement of expanded polytetrafluoroethylene (e-PTFE) sutures as replacement neochordae on a beating heart, without the need for cardiopulmonary bypass (39).

A relatively short learning curve is needed to achieve expertise in performing the NeoChord procedure safely (40). This contributed to the successful European experience and to the diffusion of the procedure so that, since its first application, more than 1,000 patients have been treated (41, 42). As for conventional surgery, the ideal candidates for NeoChord implantation are patients with isolated central posterior leaflet prolapse or flail, and patients with posterior multi-segment involvement. On the contrary, treating more complex lesions, such as those involving the anterior leaflet and paracommissural or calcified leaflets, has worse outcomes (43). Another predictor of success after NeoChord implantation is the Leaflet-to-Annulus Index (LAI), which is the ratio between the sum of the anterior leaflet length and the posterior leaflet length over the antero-posterior length. LAI values of ≤1.35, 1.30, and 1.25 are a positive prognostic predictor of residual regurgitation at 3, 6 months, and 1 year, respectively (44). According to these measurements, it is estimated that ~25–30% of patients presenting with DMR can be effectively treated with the NeoChord procedure (40). To expand its application, one possibility is the synergic combination of this device with other transcatheter MVr systems, mimicking what already currently occurs with surgery. Otherwise, the group of Colli described another interesting application of the technique, as they reproduced the edge-to-edge intervention directly through transapical neochordal implantation with satisfactory results (45).

A currently ongoing multicenter, randomized trial (NCT02803957) comparing the NeoChord procedure with conventional surgical MVr in the United States will help provide further insights on the procedure.

### Mitral Butterfly

The mitral Butterfly system (Angel Valve, Wien, Austria) aims to reproduce the butterfly repair technique for the treatment of the posterior MV leaflet prolapse. The butterfly repair consists of the combination of a triangular resection from the prolapsing edge, with a reverse triangular resection to the annulus to remove redundancy (46). The Butterfly system consists of a nitinol stent with PTFE yarns and a swing arm that mimics an artificial papillary muscle. The implant can be performed both anterogradely and retrogradely using a steerable catheter. Once released, the Butterfly system contains the prolapsing posterior segment through the PTFE yarns; the position is stabilized thanks to the swing arm. The clinical development program (cOntaining prolapsing Segments to Correct mitral Regurgitation–OSCAR) is planned to start in 2019, and contemplate a first pre-clinical phase in sheep with the first in man procedure is planned in 2021 (47).

### Polares

The Polares (Palo Alto, CA, USA) solution is a new concept in the vast armamentarium of mitral valve transcatheter technologies, it is a halfway between repair and replacement. It consists of the implantation of a posterior ePTFE neoleaflet to restore coaptation with the valve's native anterior leaflet. The development of this technology still needs to undergo clinical testing. To this date, implantation has been only performed in animal models.

### MitraMaze

The MitraMaze system (Coramaze Technologies, Hilden, Germany) is a coaptation enhancer device composed of a flexible spacer, a nitinol crown and a customized delivery catheter system, which is specifically designed for the transfemoral approach. Upon release on site in the beating heart, the self-expanding implant design allows for an atraumatic anchoring in the left atrium, without the need to include adjacent myocardial tissue structures. A nitinol crown is left in the atrium and the spacer can be filled to reduce the coaptation gap between the mitral leaflets. In ovine models, the MitraMaze has demonstrated a significant reduction of the regurgitant volume (48).

### Sutra

The Sutra (Dura Biotech) hemi-valve has been studied to specifically treat MR secondary to an isolated defect of the posterior mitral valve leaflet. The hemi-valve is composed of a tri-leaflet valve mounted on a crescent shaped stent frame. The hemi-valve is designed to enhance the systolic coaptation with the native leaflets, thus reducing the regurgitation. The hemi-valve is fixed to the posterior annulus through anchors that can be adjusted, thus allowing the possibility to cinch the native valve annulus. The first generation device has been surgically implanted in animals due to the lack of an anchoring system. However, the latest version of the device has been implanted in animals using the anchoring system. The device has demonstrated good safety and efficacy in reducing the regurgitant volume under hydrostatic testing (49).

## Conclusions

Percutaneous MVr is a rapidly growing field, with several devices at different stages of development. Due to their capacity to preserve the complex inner anatomy of the mitral valve, these repair systems will have an important role in the treatment of MR. Large clinical cohort studies will help identify the right patient population that would benefit the most from transcatheter MVr. This breakthrough of new repair devices will enlarge the percutaneous armamentarium of MVr, offering a wide possibility of treatments customizable to specific anatomical features.

## Author Contributions

AM, ALar, FGi, and FGa produced a first draft of the manuscript. FK, ALad, and LT did a review of the literature. AC and ALat reviewed the article and gave their intellectual contribution to the manuscript.

### Conflict of Interest

AM received an institutional grant (unrestricted grant) from Boston Scientific; he received a research grant from Innovative Cardiac Solution. FGi serves as proctor for Neovasc, ALat has served on advisory boards for Medtronic and Abbott; and has been a consultant to Edwards Lifesciences. The remaining authors declare that the research was conducted in the absence of any commercial or financial relationships that could be construed as a potential conflict of interest.

## Abbreviations

MVr: mitral valve repair
MR: mitral regurgitation
FMR: functional mitral regurgitation
DMR: degenerative mitral regurgitation
TMVR: transcatheter mitral valve replacement
LVEF: left ventricular ejection fraction
MAC: mitral annular calcification.
